# Identification of N6-Methylandenosine-Related lncRNAs for Subtype Identification and Risk Stratification in Gastric Adenocarcinoma

**DOI:** 10.3389/fonc.2021.725181

**Published:** 2021-09-27

**Authors:** Yuancheng Huang, Zehong Yang, Chaoyuan Huang, Xiaotao Jiang, Yanhua Yan, Kunhai Zhuang, Yi Wen, Fengbin Liu, Peiwu Li

**Affiliations:** ^1^ First Clinical Medical College, Guangzhou University of Chinese Medicine, Guangzhou, China; ^2^ Department of Gastroenterology, Baiyun Branch of the First Affiliated Hospital of Guangzhou University of Chinese Medicine, Guangzhou, China; ^3^ Department of Gastroenterology, The First Affiliated Hospital of Guangzhou University of Chinese Medicine, Guangzhou, China

**Keywords:** gastric adenocarcinoma, N6-methyladenosine, long noncoding RNAs, prognostic signature, tumor microenvironment, immunotherapy

## Abstract

**Objectives:**

The purpose of this study was to investigate the role of m^6^A-related lncRNAs in gastric adenocarcinoma (STAD) and to determine their prognostic value.

**Methods:**

Gene expression and clinicopathological data were obtained from The Cancer Genome Atlas (TCGA) database. Correlation analysis and univariate Cox regression analysis were conducted to identify m^6^A-related prognostic lncRNAs. Subsequently, different clusters of patients with STAD were identified *via* consensus clustering analysis, and a prognostic signature was established by least absolute shrinkage and selection operator (LASSO) Cox regression analyses. The clinicopathological characteristics, tumor microenvironment (TME), immune checkpoint genes (ICGs) expression, and the response to immune checkpoint inhibitors (ICIs) in different clusters and subgroups were explored. The prognostic value of the prognostic signature was evaluated using the Kaplan-Meier method, receiver operating characteristic curves, and univariate and multivariate regression analyses. Additionally, Gene Set Enrichment Analysis (GSEA), Kyoto Encyclopedia of Genes and Genomes (KEGG) pathway, and Gene Ontology (GO) analysis were performed for biological functional analysis.

**Results:**

Two clusters based on 19 m^6^A-related lncRNAs were identified, and a prognostic signature comprising 14 m^6^A-related lncRNAs was constructed, which had significant value in predicting the OS of patients with STAD, clinicopathological characteristics, TME, ICGs expression, and the response to ICIs. Biological processes and pathways associated with cancer and immune response were identified.

**Conclusions:**

We revealed the role and prognostic value of m^6^A-related lncRNAs in STAD. Together, our finding refreshed the understanding of m^6^A-related lncRNAs and provided novel insights to identify predictive biomarkers and immunotherapy targets for STAD.

## Introduction

Globally, gastric cancer (GC) is the fifth most common cancer and the third most deadly neoplasm (1). Gastric adenocarcinoma (STAD) is the most common pathological type of GC, and despite considerable progress in the diagnosis and therapeutic strategies for STAD, the prognosis of patients with STAD remains poor due to advanced stage and postsurgical recurrence (2, 3). Therefore, the identification of novel biomarkers for early detection and effective therapeutic targets for treating patients with STAD is critical and urgent.

Accumulating evidence has shown that long noncoding RNAs (lncRNAs) had various biological functions and played a crucial role in the oncogenesis and progression of GC (4). For example, lncRNA IGF2-AS functions as a competing endogenous RNA (ceRNA) to miR-503 and promotes the pathogenicity of STAD by regulating SHOX2 (5). LINC00707 acts as an oncogene in GC by interacting with RNA-binding protein HuR and increasing the stability of VAV3/F11R mRNAs (6). LncRNA CRNDE could bind to splicing protein SRSF6 to reduce its stability and thus regulate alternative splicing events and affect autophagy regulation in GC (7).

Increasing evidence suggests that RNA modifications play a critical role in tumorigenesis and progression of different cancers, including GC (8, 9). N6-methyladenosine (m^6^A), which introduces a methyl group in the nitrogen-6 position of adenosine, is found to be the most frequent internal RNA modification in mammals (10). As a dynamic and reversible process, m^6^A RNA modification is primarily regulated by “writers” (adenosine methyltransferases) and “erasers” (demethylases) and performs different functions by interacting with “readers” (m^6^A-binding proteins). As identified to distribute extensively in a variety of RNAs, such as messenger RNAs (mRNAs), pri-microRNAs (pri-miRNAs), circular RNAs (circRNAs), and lncRNAs, m^6^A is involved in various biological processes related to the occurrence and progression of tumors, including GC (11–13). For instance, the m^6^A writer METTL3 stimulates m^6^A modification of HDGF mRNA, and the m^6^A reader IGF2BP3 recognizes and binds to the m^6^A site and enhances its stability, which promotes tumor angiogenesis and glycolysis in GC (14). Overexpression METTL3 facilitates the processing of pri-miR-17 into the miR-17 through an m^6^A DGCR8-dependent method, which activates the AKT/mTOR pathway and the progression of GC (15). LINC00470 promotes the degradation of PTEN mRNA to facilitate malignant behavior in GC cells by interacting with METTL3 (16). Additionally, extensive literature has demonstrated that m^6^A plays an important role in immune recognition, immune responses, and tumor microenvironment (TME) (17–19). However, the specific role and prognostic value of m^6^A-related lncRNAs in STAD remain unclear.

Here, we analyzed the Cancer Genome Atlas (TCGA) database for m^6^A-related lncRNAs involved in STAD, identified two clusters based on m^6^A-related lncRNAs, and constructed an m^6^A-related lncRNA prognostic signature. Then, we estimated its predictive value and diagnostic effectiveness, as well as the correlation of m^6^A-related lncRNAs with TME and immunotherapy. Furthermore, the molecular mechanisms associated with m^6^A-related prognostic lncRNAs were explored. The finding in this study revealed the critical role of m^6^A-related lncRNAs and shed light on the latent relationship and the underlying mechanism between m^6^A-related lncRNAs and tumor-immune interactions.

## Material and Methods

### Acquisition of Datasets

The RNA-seq transcriptome data [fragments per kilobase million (FPKM)] (20) from 373 samples and clinical information from 406 patients with STAD in the TCGA database (http://cancergenome.nih.gov/) were downloaded for our study. Patients with complete clinicopathological and survival information were included for further assessment.

### Selection of m^6^A-Related Regulators

Based on published data (21–23), 24 m^6^A-related regulators, including METTL3, METTL14, METTL16, WTAP, VIRMA, KIAA1429, ZC3H13 RBM15, RBM15B, YTHDC1, YTHDC2, YTHDF1, YTHDF2, YTHDF3, HNRNPC, FMR1, LRPPRC, HNRNPA2B1, IGFBP1, IGFBP2, IGFBP3, RBMX, FTO, and ALKBH5, were used in our study.

### Bioinformatic Analysis

Primarily, the correlation analysis was performed between m^6^A-related regulators and all lncRNAs in STAD. m^6^A-related lncRNAs were identified based on the following classification parameters (1): correlation coefficients more than 0.4 and (2) *p*-value less than 0.001. Then, to filtrate the m^6^A-related lncRNAs that were highly correlated with overall survival (OS), univariate Cox regression analysis was performed. Additionally, the correlation analysis of m^6^A-related prognostic lncRNAs was implemented using the “corrplot” package in R. Next, to explore the potential function of m^6^A-related lncRNAs in STAD, two different clusters (clusters I and II) were identified using the “Consensus ClusterPlus” R package (24) based on the expression of m^6^A-related prognostic lncRNAs with a resample rate of 80%, 50 iterations, and Pearson’s correlation. The different clinicopathological characteristics and OS were compared between clusters I and II. Furthermore, the differences in the content of immune infiltrating cells, TME scores, and the expression of immune checkpoint genes (ICGs) between different clusters were explored (25). The content of immune infiltrating cells was identified by CIBERSORT (26), and the immune/stromal scores and tumor purity were calculated through the “ESTIMATE” package in R (27).

Then, we randomly divided the patients with STAD into two groups: the training group and the testing group. Subsequently, based on m^6^A-related prognostic lncRNAs identified by univariate Cox regression analysis, the least absolute shrinkage and selection operator (LASSO) Cox regression algorithm was used to identify m^6^A-related lncRNAs with powerful prognostic significance and construct the prognostic risk model from the training group data. According to the best penalty parameter *λ*, the coefficients of the m^6^A-related lncRNAs were calculated. The risk score (RS) was estimated using the following formula:


$$\text{RS}=\sum_{i=1}^{n}\text{Coef}(i)X(i)$$

where Coef(i) is the coefficient and *X*(*i*) represents the expression levels of m^6^A-related lncRNAs. Using the median RS obtained as the demarcation value, patients with STAD were classified into two groups: high-risk and low-risk groups. Kaplan-Meier analysis and the receiver operating characteristic (ROC) curves were used to validate the predictive efficiency (28). Then, the accuracy of the model was validated from the test group and the combined group using the same method. Furthermore, the differences in clinicopathological features, the content of immune infiltrating cells, TME scores, and ICGs expression between high-risk and low-risk groups were also explored. Moreover, we further predicted the response to immune checkpoint inhibitors (ICIs) in subgroups based on the immunophenoscores (IPS) of patients with STAD obtained from The Cancer Immunome Atlas (TCIA) (https://tcia.at/home). Additionally, the prognostic value of the RS was verified using univariate and multivariate Cox regression analyses. The hazard ratio (HR) with 95% confidence intervals and log-rank *p*-value were calculated using the “glmnet” and “survival” R packages (29).

**Figure 1:**
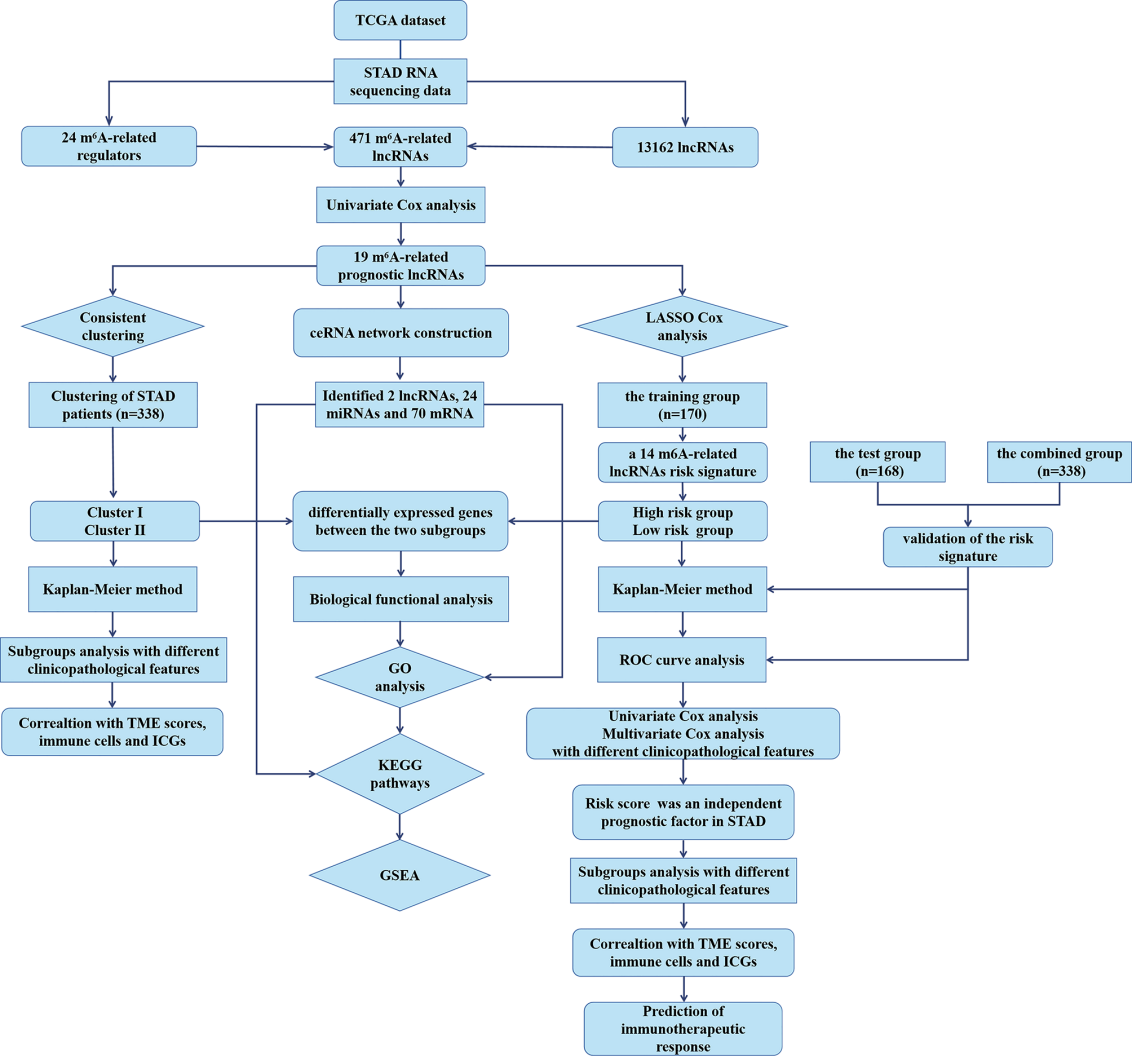
The flow chart of the study design and analysis.

To explore the biological functions associated with m^6^A-related lncRNAs, Gene Set Enrichment Analysis (GSEA), Kyoto Encyclopedia of Genes and Genomes (KEGG) pathway, and Gene Ontology (GO) analysis were performed. Genes that were significantly upregulated (fold change >1 and *p* < 0.05) or downregulated (fold change <−1 and *p* < 0.05) between clusters I and II or between the high-risk and low-risk groups were identified using the “edgeR” package in R, which were used for GO and KEGG pathway analysis. Additionally, genes in different clusters and different risk groups were functionally annotated using GSEA. Based on the m^6^A-related prognostic lncRNAs, the target miRNAs were predicted *via* miRcode database and target mRNAs of these miRNAs were found in different databases, such as TargetScan, miRTarBase, and miRDB. Target mRNAs in the ceRNA network were also functionally annotated using GO and KEGG pathway analyses. The flow chart of bioinformatic analysis was shown in **Figure 1**.

### Cell Culture

The GC cell line MGC-803 and the normal human gastric epithelial cell line GES-1 were purchased from the American Type Culture Collection (ATCC, Manassas, VA, USA). All cells were cultured in RPMI-1640 medium (Life Technologies, Grand Island, NY, USA) supplemented with 10% fetal bovine serum (Life Technologies) at 37°C in a humidified atmosphere with 5% CO_2_.

### Quantitative Reverse Transcription-Polymerase Chain Reaction

Total RNA was extracted from cells with TRIzol reagent (Invitrogen, China) according to manufacturer’s instruction. Reverse transcription was carried out according to the manufacturer’s instructions using the PrimeScript RT Reagent Kit (Takara, China). The SYBR PrimeScript RT-PCR Kit (Takara) was applied for the analysis of quantitative reverse transcription-polymerase chain reaction (qRT-PCR). Related lncRNAs expression levels were calculated using the 2-ΔΔCT method and the related GAPDH mRNA expression was used as an endogenous control. Primers sequences used in our study were as follows: GAPDH forward 5′-GGACCTGACCTGCCGTCTAG-3′, and reverse 5′-GTAGCCCAGGATGCCCTTGA-3′; SREBF2-AS1 forward 5′-TAGTGCCGCTGCTGGAAA-3′, and reverse 5′-TGTGGGAGTCGTGCTGGT-3′; LINC00106 forward 5′-AAGCATTTGGCAAGCACA-3′, and reverse 5′-GCCTGAAGTCTTCCCGTTA-3′; SENCR forward 5′-CCACGCTTTGGACTTGCT-3′, and reverse 5′-GCGGGTTTCTGGTGAGGT-3′; LINC01537 forward 5′-GTCGGGATACATCTTGGT-3′, and reverse 5′-TTGAGTTGTTCTGCCTTT-3′; MAGI2-AS3 forward 5′-CCTTACTTCTAGGCTTCT-3′, and reverse 5′-GTTTACTTTGCTGGTGTC-3′; STARD4-AS1 forward 5′-TCAAACAAGTATTCACCCTA-3′, and reverse 5′-ATCACCCATTCTCCACAT-3′.

### Statistical Analysis

The expression data of m^6^A-related regulators and all lncRNAs in tumor tissues and adjacent mucosa of STAD obtained from TCGA was compared using one-way analysis of variance (ANOVA); the clinical characteristics of different groups were compared using the Chi-square test; the Kaplan-Meier method was used to perform a bilateral logarithmic rank test in overall survival analysis; *p*-value <0.05 were regarded as statistically significant. All statistical analyses were implemented using R v4.0.3 (https://www.r-project.org/) or GraphPad Prism software (Version 8.0).

## Results

### Identification of m^6^A-Related Prognostic lncRNAs

**Figure 2:**
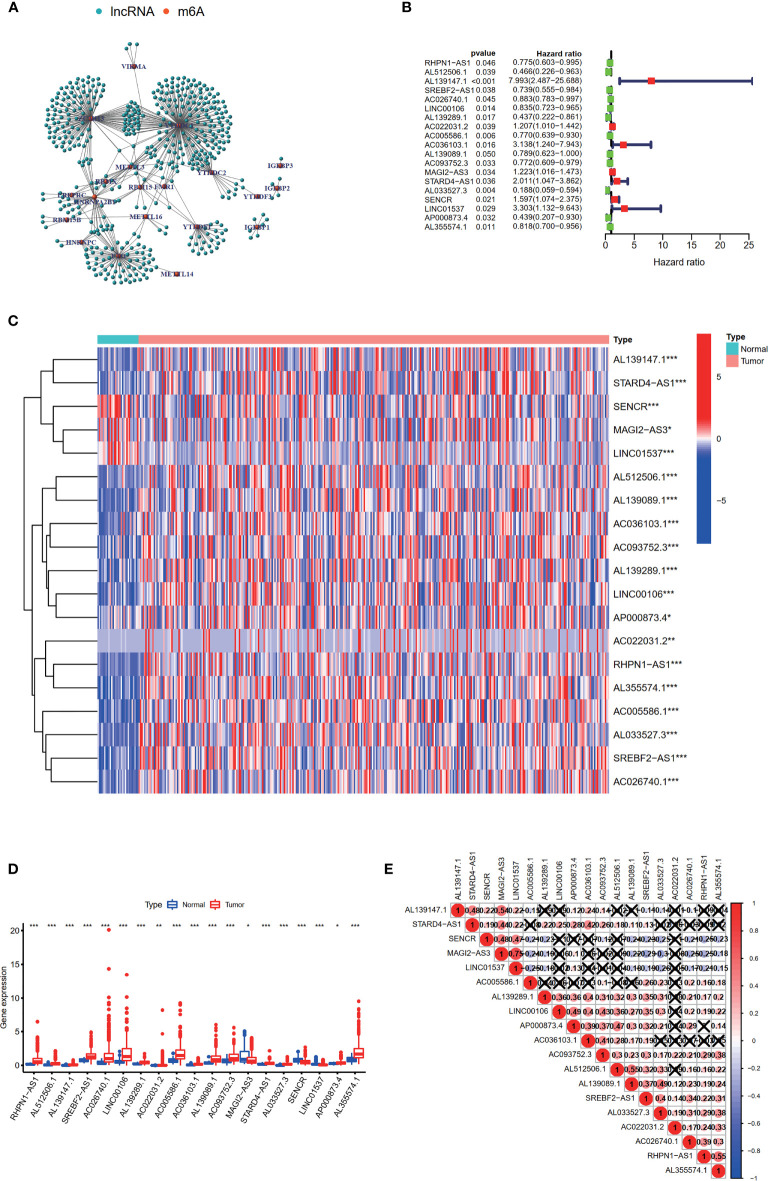
Identification of m^6^A-related prognostic lncRNAs in STAD patients. **(A)** The network of the 24 m^6^A-related regulators and 471 m^6^A-related lncRNAs. **(B)** The hazard ratio (HR) and 95% confidence interval (CI) of 19 m^6^A-related lncRNAs estimated by univariate Cox regression. **(C, D)** The expression of 19 prognostic m^6^A-related lncRNAs in TCGA database between the tumor group and the normal group. **(E)** Spearman’s correlation analysis of the 19 m^6^A-related prognostic lncRNAs. *p < 0.05, **p < 0.01, and ***p < 0.001.

Firstly, the expression levels of 24 m^6^A-related genes and all lncRNAs from the TCGA were extracted respectively. Through coexpression analysis, we identified 471 m^6^A-related lncRNAs (|cor| > 0.4, *p*-value <0.05). The gene co-expression network of 24 m^6^A-related genes and 471 m^6^A-related lncRNAs was shown in **Figure 2A**. After conducting univariate Cox analysis, 19 candidate lncRNAs that were highly correlated with OS were identified (*p* < 0.05) (**Figure 2B**). The expression of 19 m^6^A-related prognostic lncRNAs was compared between tumor tissues and adjacent mucosa (**Figures 2C, D** **)**. Among these lncRNAs, seven (AL139147.1, AC022031.2, AC036103.1, MAGI2-AS3, STARD4-AS1, SENCR, LINC01537) were prognostic risk factors, and 12 (RHPN1-AS1, AL512506.1, SREBF2-AS1, AC026740.1, LINC00106, AL139289.1, AC005586.1, AL139089.1, AC093752.3, AL033527.3, AP000873.4, AL355574.1) were prognostic protective factors. The 19 m^6^A-related lncRNAs were closely correlated with each other (**Figure 2E**).

### Consensus Clustering of m^6^A-Related Prognostic lncRNAs Identified Two Clusters of STAD With Different Clinicopathological Features and Immune Landscape

**Figure 3:**
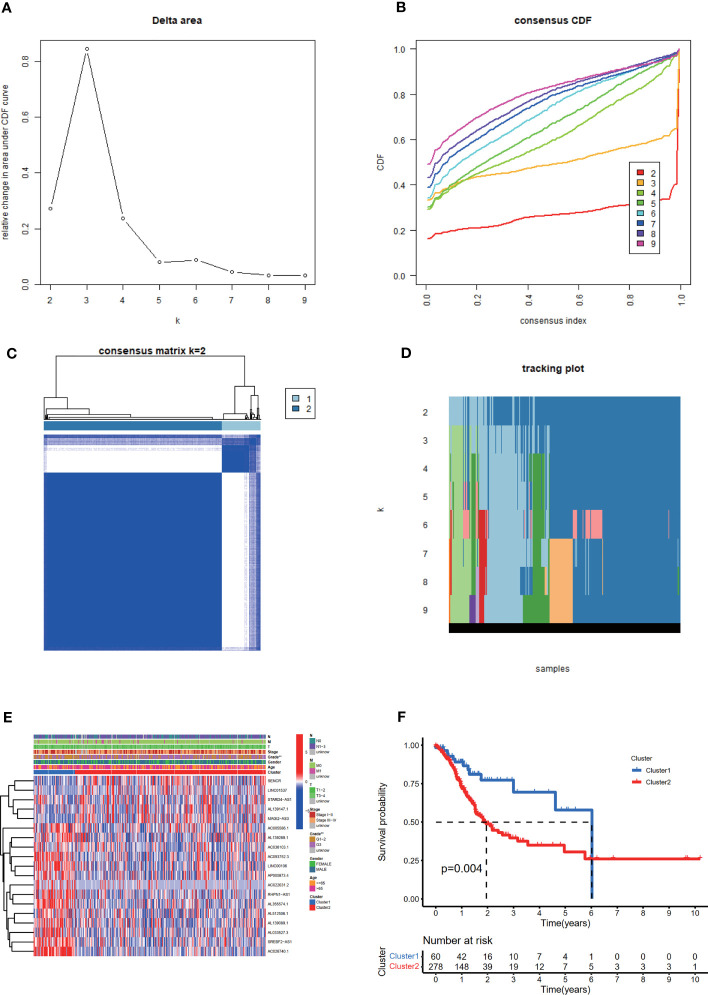
Consistent cluster analysis of patients with STAD based on 19 prognostic m^6^A-related lncRNAs. **(A)** The consistency clustering cumulative distribution function (CDF) when *k* is between 2 and 10. **(B)** The relative change of the area under the CDF curve from 2 to 10 of *k* **(C)** At *k* = 2, the correlation between groups. **(D)** The distribution of the sample when *k* is between 2 and 10. **(E)** The distribution of clinicopathological characteristics and the expression of 19 prognostic m^6^A-related lncRNAs in clusters I and II. **p < 0.01. **(F)** Comparison of Kaplan-Meier overall survival (OS) curve for STAD patients in clusters I and II.

Based on the expression levels of 19 m^6^A-related prognostic lncRNAs, consistent clustering analysis of patients with STAD was implemented. Patients were clustered into two clusters due to the minimal interference between the two subgroups **(** **Figures 3A–D** **)**.

The distribution of the clinicopathological characteristics in clusters I and II were displayed as a heat map **(** **Figure 3E** **)**. Evident differences between the two clusters according to tumor grade (*p* < 0.01) were observed. Notably, the OS rate of clusters I and II were significantly different based on the Kaplan-Meier method and cluster II was associated with poorer OS **(** **Figure 3F** **)**.

**Figure 4:**
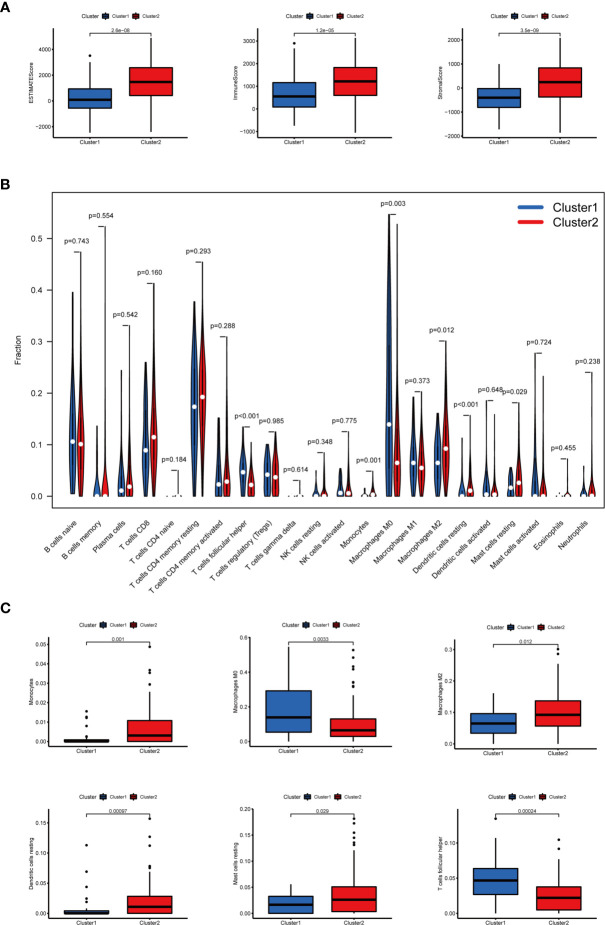
The TME scores and the content of 22 immune cells in clusters I and II. **(A)** The ESTIMATE, immune, and stromal scores significantly increased in cluster II. **(B)** The content of 22 immune cells between clusters I and II. **(C)** The infiltration status of follicular helper T cells, M0 macrophages, M2 macrophages, monocytes, resting dendritic cells, and resting mast cells between cluster I and cluster II was significantly different.

In terms of TME scores, it was found that ESTIMATE, immune, and stromal scores significantly increased in cluster II, while tumor purity increased in cluster I. In addition, the content of 22 immune cells in clusters I and II was compared **(** **Figure 4A** **)**. As a result, cluster I contained more follicular helper T cells (*p* < 0.001) and M0 macrophages (*p* < 0.01), and cluster II had more monocytes (*p* < 0.01), M2 macrophages (*p* < 0.05), resting dendritic cells (DC) (*p* < 0.001) and resting mast cells(*p* < 0.05). Differential analysis of immune infiltration cells was displayed in **Figures 4B, C**.

**Figure 5:**
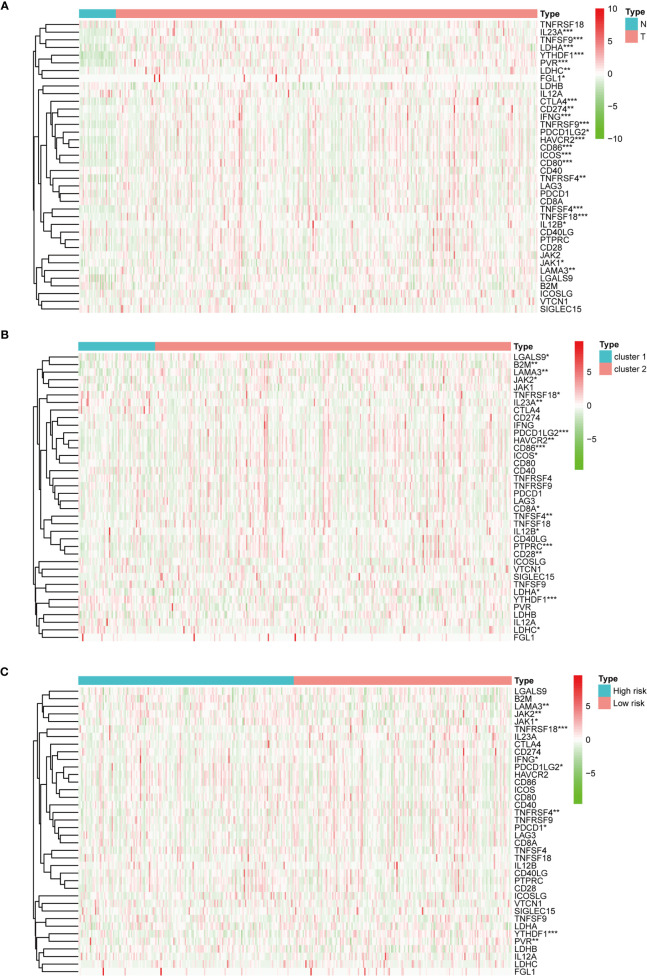
The expression of immune checkpoint genes. **(A)** The expression of immune checkpoint genes between the normal and the tumor groups. **(B)** The expression of immune checkpoint genes between cluster I and cluster II. **(C)** The expression of immune checkpoint genes between the high-risk and the low-risk groups. *p < 0.05, **p < 0.01, and ***p < 0.001.

Regarding the expression of ICGs, we investigated the distribution of 38 ICGs obtained from previous studies in different clusters (30–34). Through differential expression analysis, we found that 22 ICGs in the tumor tissues differentially expressed compared with the adjacent mucosa **(** **Figure 5A** **)**, and 11 of 22 ICGs in cluster I differentially expressed compared with cluster II **(** **Figure 5B** **)**. Moreover, we observed three ICGs (namely, YTHDF1, IL23A, LDHC) were significantly overexpressed in cluster I and eight ICGs (namely, PDCD1LG2, CD86, HAVCR2, LAMA3, TNFSF4, IL12B, LDHA, ICOS) were significantly overexpressed in cluster II.

### Construction and Verification of the m^6^A-Related lncRNAs Prognostic Signature

**Figure 6:**
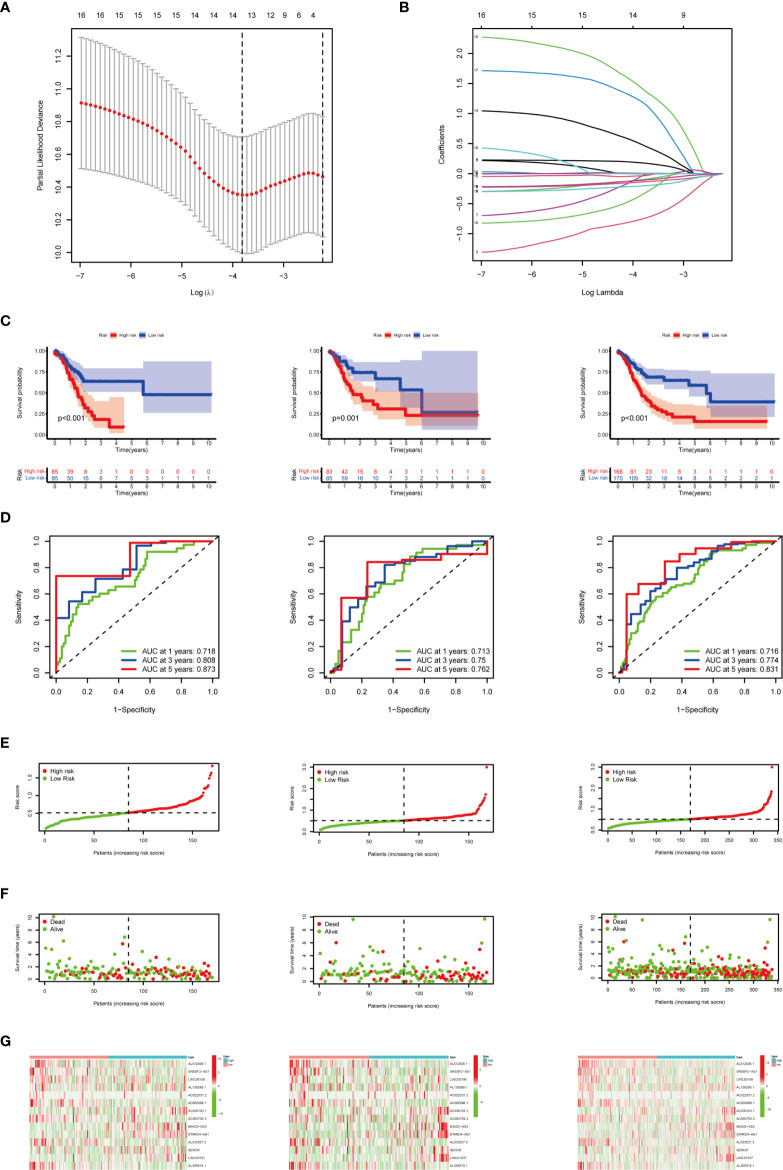
Construction and verification of the m^6^A-related lncRNA prognostic signature. **(A)** The point with the smallest cross-verification error corresponds to the number of factors included in the LASSO regression model. **(B)** The lines of different colors represent the trajectory of the correlation coefficient of different factors in the model with the increase of Log Lamda. **(C)** Kaplan-Meier analysis of patients in the high-risk and low-risk groups in the training group, the test group, and the combined group. **(D)** ROC analysis of 1, 3, and 5 years in the training group, the test group, and the combined group. **(E)** Distribution of patients with different risk scores in the training group, the test group, and the combined group. **(F)** OS status of patients with different risk scores in the training group, the test group, and the combined group. **(G)** Heat map of the prognostic signature scores in the training group, the test group, and the combined group.

Based on 19 candidate lncRNAs that were highly correlated with OS, we used the LASSO method in the training group to construct an m^6^A-related lncRNA signature for evaluating the prognosis of patients with STAD. Finally, 14 lncRNAs were chosen to establish a prognostic signature and the risk score was calculated **(** **Figures 6A, B** **)**. Using the median risk score as the demarcation value, the patients in the training group (*n* = 170) were classified into two groups, namely, the high-risk and low-risk groups. To test the efficacy of the prognostic model, survival and ROC curve analyses were conducted. Kaplan-Meier analysis showed that the low-risk group had a significantly longer survival time than the high-risk group (*p* < 0.001) **(** **Figure 6C** **)**. The value of the area under the curve (AUC) in the time-dependent ROC curve of 1, 3, and 5 years was 0.718, 0.808, and 0.873 severally **(** **Figure 6D** **)**, suggesting good prediction performance of the survival model. To further validate this 14 lncRNA prognostic signature, verification analysis in the test group (*n* = 168) and the combined group (*n* = 338) was implemented. As a result, the high-risk group in the test group and the combined group had significantly shorter survival time compared with the low-risk group, which was previously observed in the training group **(** **Figure 6C** **)**. The time-dependent ROC curve of the test group and the combined group also had well-prediction performances, and the AUC value of 1, 3, and 5 years is shown in **Figure 6D**. The distribution plot of the risk score and survival status showed that the higher the risk score, the more deaths of patients with STAD (**Figures 6E, F**).The expression of 14 m^6^A-related prognostic lncRNAs in the training group, the test group and the combined group, was displayed as a heat map (**Figures 6G**).

**Figure 7:**
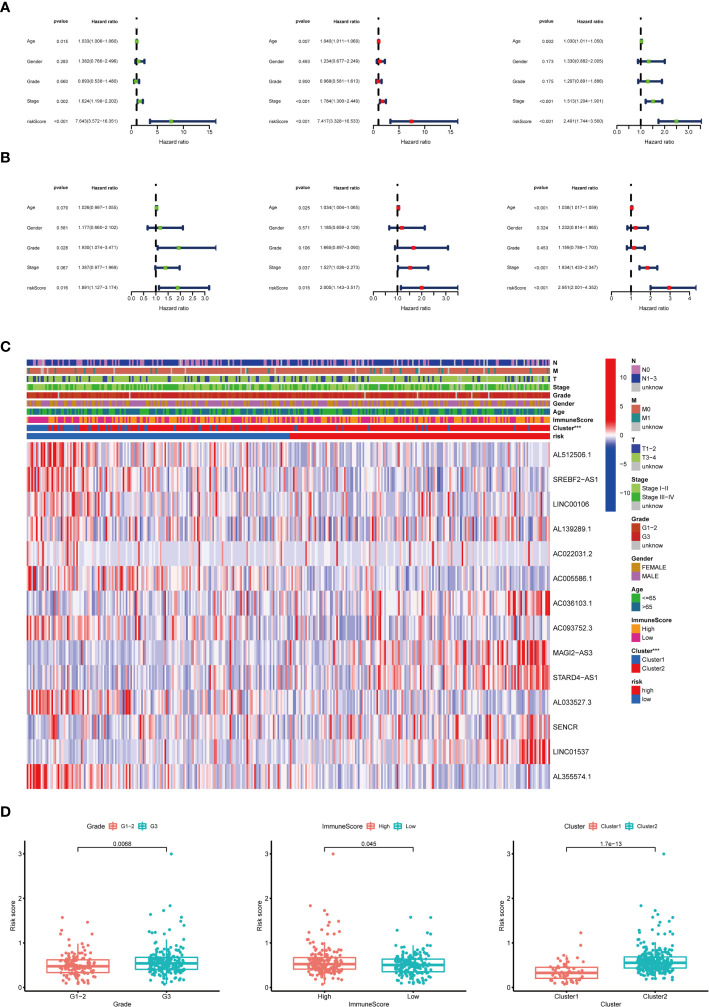
Relationship between the risk score and clinicopathological characteristics. **(A)** Univariate Cox regression analysis of the association between clinicopathological factors (including risk score) and OS of patients in the training group, the test group, and the combined group. **(B)** Multivariate Cox regression analysis of the association between clinicopathological factors (including risk score) and OS of patients in the training group, the test group, and the combined group. **(C)** The heat map showed the expression of 14 m^6^A-related prognostic lncRNAs, the distribution of clinicopathological characteristics, the immune scores of TME, and the clustering of patients in the high-risk and low-risk groups. ***p < 0.001. **(D)** The risk score was significantly higher in patients with higher-grade tumors (*p* < 0.01), higher immune scores of TME (*p* < 0.05), and cluster II (*p* < 0.01).

To examine whether the risk score was an independent prognostic factor, univariate and multivariate Cox regression analyses were conducted. In the training group, the risk score was significantly associated with OS both in univariate and multivariate Cox regression analyses (*p* < 0.001), in addition to age at diagnosis and pathological stage (*p* < 0.05). It should be noted that the risk score was also closely related with OS in the test group (*p* < 0.05) and the combined group (*p* < 0.001) by the same analysis, which indicated that the risk score was an independent powerful prognostic factor for the prognosis of OS in STAD **(** **Figures 7A, B** **)**.

### Subgroup Analysis With Different Clinicopathological Features and Immune Landscape

The expression of 14 m^6^A-related prognostic lncRNAs and the distribution of clinicopathological characteristics, the immune scores of TME, and the clustering of patients in the high-risk and low-risk groups were displayed as a heat map **(** **Figure 7C** **)**. Evident differences between the two groups according to different clusters (*p* < 0.001) were observed. Significant differences of risk score were found between (1): different tumor grades (*p* < 0.01) (2), different immune scores of TME (*p* < 0.05), and (3) different clusters (*p* < 0.01) **(** **Figure 7D** **)**.

**Figure 8:**
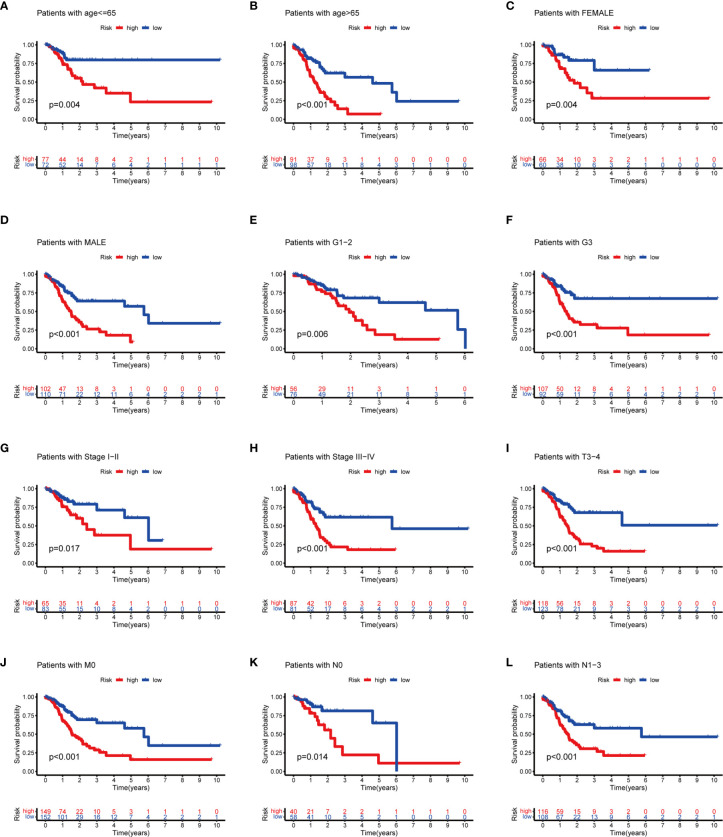
Subgroup analysis with different clinicopathological features in STAD: **(A)** age ≤65; **(B)** age >65; **(C)** female; **(D)** male; **(E)** G1–G2; **(F)** G3; **(G)** stages I–II; **(H)** stage III–IV; **(I)** T3–T4; **(J)** M0; **(K)** N0; and **(L)** N1.

To evaluate whether the m^6^A-related lncRNAs prognostic model could serve as a prognostic indicator for OS in subgroups of patients with different clinical characteristics, we stratified subgroups by age (age ≤65 and age >65), gender (female and male), grade (G1–G2 and G3), clinical stage (stages I–II and III–IV), stage T (T1–T2 and T3–T4), stage M (M0 and M1), and stage N (N0 and N1). As the result shown in **Figure 8**, the OS of the low-risk patients based on age (*p* = 0.004 in age ≤65 and *p* < 0.001 in age >65), sex (*p* = 0.004 in female and *p* < 0.001 in male), grade (*p* = 0.006 in G1–G2 and *p* < 0.001 in G3), clinical stage (*p* = 0.017 in stages I–II and *p* < 0.001 in stages III–IV), stage T3–T4 (*p* < 0.001), stage M0 (*p* < 0.001), and stage N (*p* = 0.014 in N0 and *p* < 0.001 in N1) was significantly higher than those of the high-risk patients.

**Figure 9:**
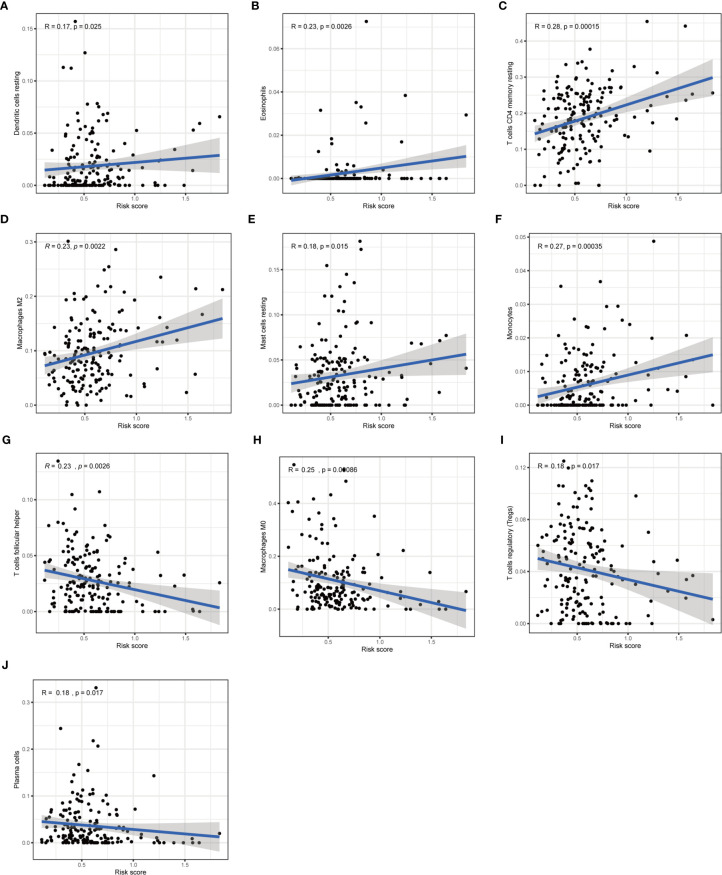
Correlation of subgroup with immune infiltration level in STAD. **(A–F)** The high-risk group has significant positive correlations with resting DC, eosinophils, memory resting CD4 T cells, M2 macrophages, resting mast cells, and monocytes. **(G–J)** The high-risk group has significant negative correlations with M0 macrophages, plasma cells, follicular helper T cells, and regulatory T cells.

**Figure 10:**
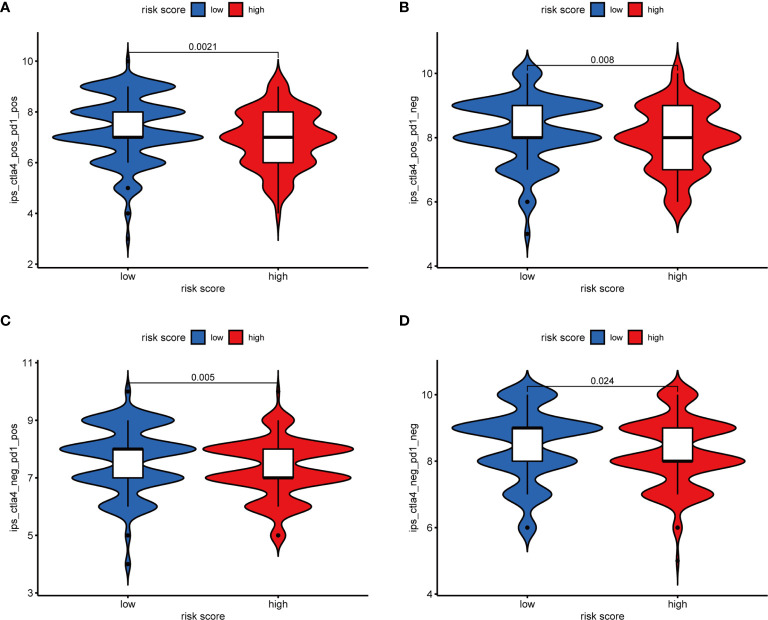
The relative probabilities to respond to anti-PD-1/PD-L1 and anti-CTLA-4 treatment in the low-risk score and high-risk score group. **(A)** IPS-PD1(+)/CTLA4(+). **(B)** IPS-PD1(−)/CTLA4(+). **(C)** IPS-PD1(+)/CTLA4(−). **(D)** IPS-PD1(−)/CTLA4(−).

Furthermore, we analyzed the association between immune cells and ICGs and subgroups. As a result, we found that the high-risk group had significant positive correlations with infiltrating levels of resting DC (*r* = 0.17, *p* = 0.025), eosinophils (*r* = 0.23, *p* = 0.0026), M2 macrophages (*r* = 0.23, *p* = 0.0022), resting mast cells (*r* = 0.18, *p* = 0.015), monocytes (*r* = 0.27, *p* = 0.00035), memory resting CD4 T cells (*r* = 0.28, *p* = 0.00015) **(** **Figures 9A–F** **)**, and the low-risk group had significant positive correlations with infiltrating levels of M0 macrophages (*r* = −0.25, *p* = 0.00086), plasma cells (*r* = −0.18, *p* = 0.017), follicular helper T cells (*r* = −0.23, *p* = 0.0026), regulatory T cells (*r* = −0.18, *p* = 0.017) **(** **Figures 9G–J** **)**. Next, we observed that four ICGs (namely, JAK2, LAMA3, PDCD1LG2, JAK1) were significantly high expression in high-risk group and six ICGs (namely, YTHDF1, TNFRSF18, TNFRSF4, PVR, PDCD1, IFNG) were significantly high expression in low-risk group **(** **Figure 5C** **)**. Furthermore, the difference in the response of ICIs between high-risk and low-risk groups was explored. As a result, the low-risk group was more likely to respond to immunotherapy than the high-risk group whether they were compared in IPS-PD1(+)/CTLA4(+), IPS-PD1(+)/CTLA4(−), IPS-PD1(−)/CTLA4(+), and IPS-PD1(−)/CTLA4(−) **(** **Figure 10** **)**.

### Construction of the ceRNA Network and Functional Enrichment Analysis

**Figure 11:**
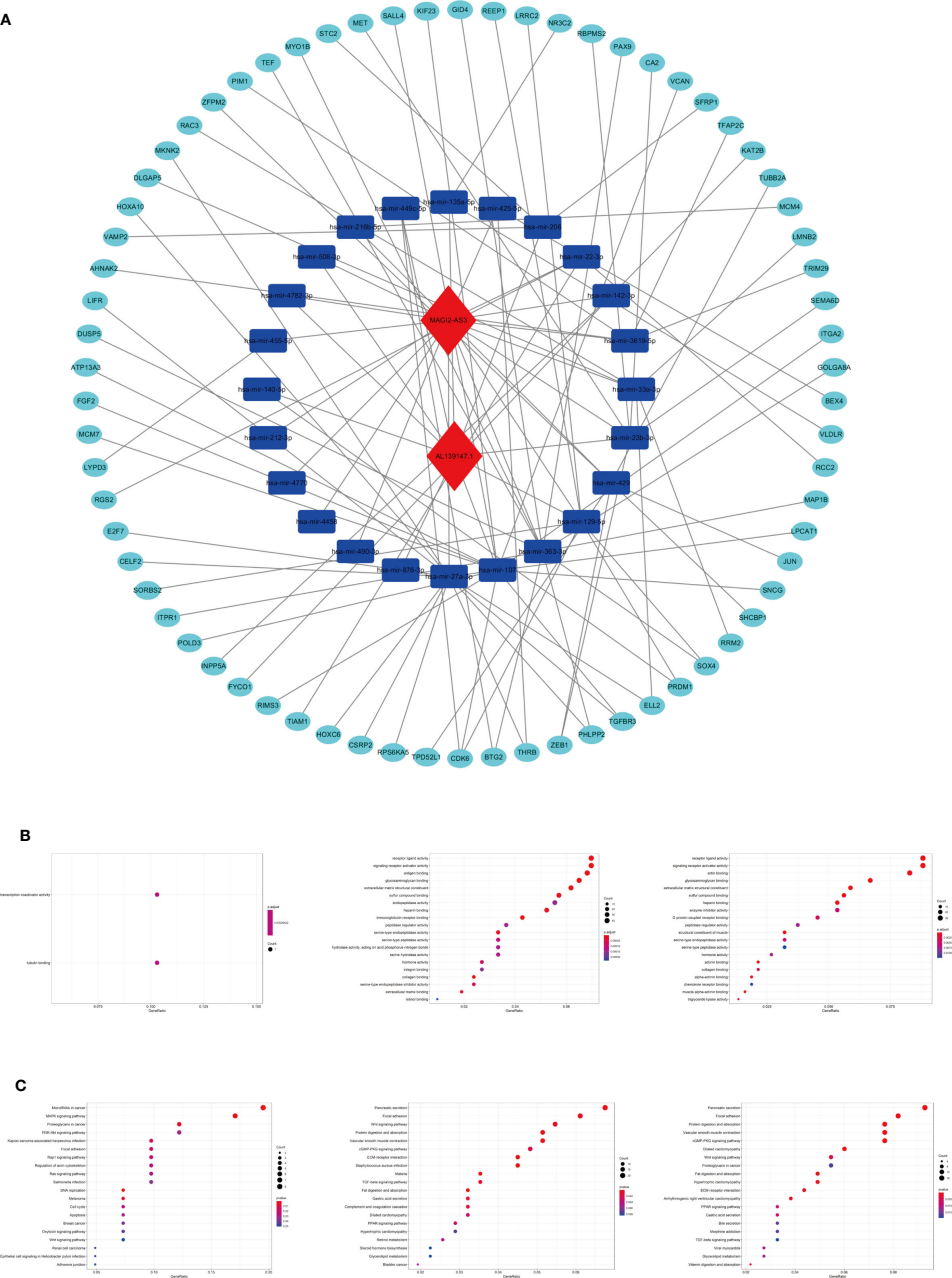
The ceRNA network construction and biological function analysis. **(A)** The ceRNA network of two lncRNAs (red) and their target miRNAs (blue) and mRNAs (green). **(B)** GO analysis in 70 target mRNAs, differentially expressed genes between cluster I and cluster II, differentially expressed genes between the high-riska and the low-risk group. **(C)** KEGG pathway analysis in 70 target mRNAs, differentially expressed genes between cluster I and cluster II, differentially expressed genes between the high-risk group and the low-risk group.

To explore the biological function of 19 m^6^A-related prognostic lncRNAs, a ceRNA network was constructed based on the mechanism of lncRNAs regulating mRNAs expression by sponging miRNAs. Two lncRNAs were extracted from the miRcode database and 28 pairs of interaction between the Two lncRNAs and 24 miRNAs were identified. Based on three mRNA predicting databases mentioned previously and differentially expressed mRNA between the normal and tumor groups of STAD in TCGA, we identified 70 target mRNA. Finally, two lncRNAs, 24 miRNAs, and 70 mRNAs were included to construct ceRNA by Cytoscape software 3.7.1 **(** **Figure 11A** **)**. KEGG pathways and GO analysis were performed to annotate the function of 70 target mRNAs, and we found that these target mRNAs were enriched in transcription coactivator activity and tubulin binding (GO analysis); microRNAs in cancer, MAPK signaling pathway, proteoglycans in cancer, PI3K-Akt signaling pathway, focal adhesion, Rap1 signaling pathway, and Ras signaling pathway (KEGG pathways) **(** **Figures 11B, C** **)**.

As we stratified the patients with STAD into clusters I and II or high-risk and low-risk groups, genes that were significantly upregulated (fold change >1 and *p* < 0.05) or downregulated (fold change <−1 and *p* < 0.05) between the different clusters or different subgroups were identified using the “edgeR” package in R. GO and KEGG pathways analysis were used for biological functional analysis. Concerning the differentially expressed genes between different clusters, these genes were associated with immune-related biological processes, such as “antigen binding” and “immunoglobulin receptor binding,” and malignancy-associated pathways, including “focal adhesion” and “Wnt signaling pathway.” The differentially expressed genes between the high-risk and the low-risk groups were enriched in malignancy-associated biological processes and pathways, containing “chemokine receptor binding”, “chemokine activity (GO analysis)”, and “focal adhesion” and “Wnt signaling pathway (KEGG pathway) **(** **Figures 11B, C** **)**”.

**Figure 12:**
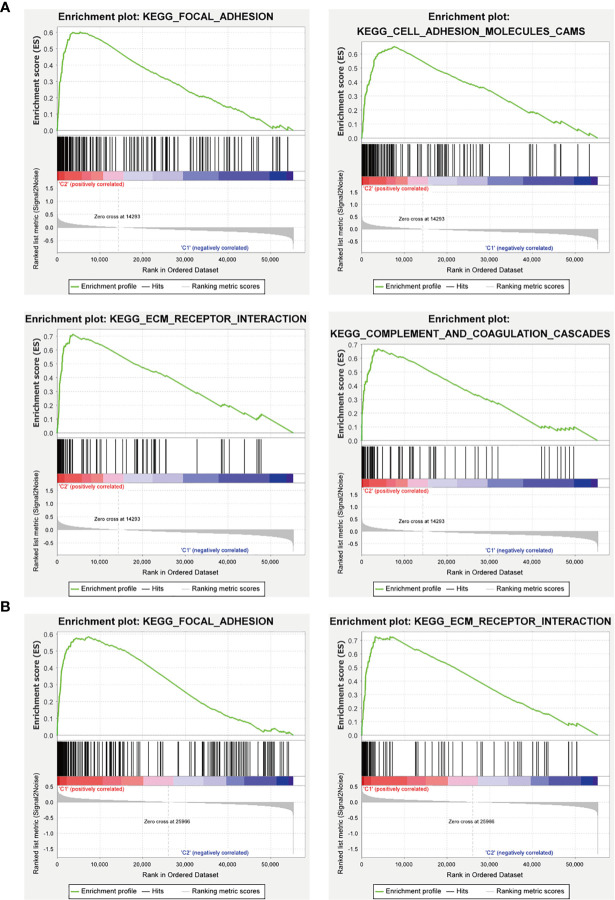
GSEA analysis of the cluster II and the high-risk group. **(A)** The cluster II was mainly enriched in “focal adhesion”, “ECM-receptor interaction”, and “cell adhesion molecules (CAMs)”, and “complement and coagulation cascades”. **(B)** The high-risk group was mainly enriched in “focal adhesion” and “ECM-receptor interaction”.

Furthermore, we used GSEA to predict the functional difference between clusters I and II or between the high-risk and the low-risk groups. The results showed that cluster II had a worse OS and a lower 5-year survival rate was closely related with the malignant hallmarks of cancer, such as “focal adhesion”, “ECM-receptor interaction”, and “cell adhesion molecules (CAMs)” and immune-related pathway, such as, “complement and coagulation cascades”. The high-risk group was also associated with cancer-related pathways, which were similar to cluster II **(** **Figure 12** **)**.

### Validation of the Expression Levels of the m^6^A-Related lncRNA in Cell Lines

**Figure 13:**
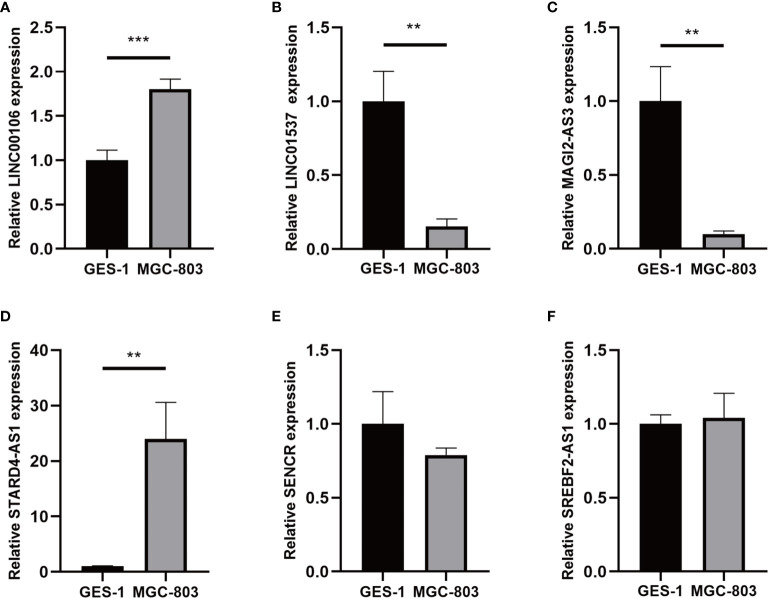
**(A–F)** Expression of six lncRNAs from the prognostic signature in GC cell line MGC-803 and normal human gastric epithelial cell line GES-1. **p < 0.01, and ***p < 0.001.

For validating the expression levels of the m^6^A-related prognostic lncRNAs from prognostic signature, we detected six m^6^A-related prognostic lncRNA expression levels in GC cell line MGC-803 and normal human gastric epithelial cell line GES-1. Our results showed that LINC01537 and MAGI2-AS3 were significantly downregulated in MGC-803 compared with GES-1 while LINC00106 and STARD4-AS1 were significantly upregulated. SREBF2-AS1 was highly expressed in MGC-803 and SENCR was lowly expressed in MGC-803, but they had no significant difference in cells **(** **Figure 13** **)**.

## Discussion

Recently, an increasing number of studies focusing on the role of lncRNAs in GC proved that lncRNAs exerted a critical oncogenic role based on their dysregulated expression and localization (35). Additionally, as the most abundant posttranscriptional modification in eukaryotic noncoding RNAs (ncRNAs), m^6^A has a huge effect on its stability and transport (36–38). Previous studies have shown that m^6^A “writers” and “erasers” could adjust the levels of m^6^A modification in mRNAs and ncRNAs to regulate binding sites to m^6^A “reader” proteins. Different m^6^A reader proteins recognize and bind to methylated ncRNAs to perform different functions. For instance, YTHDF3 recognizes and binds to m^6^A-modified lncRNA GAS5 and promotes its degradation, which elevates YAP expression and promotes colorectal cancer (CRC) progression (39). The m^6^A mark increases the stability of lncRNA FAM225A, which promotes the progression of nasopharyngeal carcinoma by acting as ceRNA to sponge miR-590-3p/miR-1275 (40). IGF2BP2 recognizes and binds to m^6^A-modified circRNA NSUN2 and increases its export to the cytoplasm (41). Overexpression METTL3 can significantly increase the nuclear localization of lncRNA RP11 in CRC cells (42). Additionally, copious studies have shown that m^6^A RNA modification affects the maturation and response function of immune cells in tumor immunity and could remodel TME (43, 44). For example, durable neoantigen-specific immunity is regulated by m^6^A-modified mRNA through YTHDF1 (45). METTL3-mediated m^6^A of CD40, CD80, and TLR4 signaling adaptor Tirap transcripts could enhance their translation in DC for stimulating T-cell activation (46). Moreover, some studies have indicated that m^6^A-modified lncRNAs participate in the immune response. As DC migration is critical for the protective immunity and immune homeostasis, m^6^A modification promotes the degradation of lncRNA Dpf3, which is associated with DC migration (47). Thus, in consideration of the crucial role of lncRNAs, m^6^A RNA modification, and immunity response in STAD, these researches call our attention to investigate the gene profile of m^6^A-related lncRNAs in STAD, explore whether m^6^A-related lncRNAs could serve as ideal biomarkers for STAD prognosis and participate in STAD initiation, progression, TME, and immunotherapy.

In our study, a total of 373 samples, 406 patients with STAD, 24 m^6^A-related regulators, and 13,162 lncRNAs were included to exploit the specific role of m^6^A-related lncRNAs in STAD. Nineteen candidate lncRNAs that were highly correlated with OS were identified. Based on the expression of 19 m^6^A-related prognostic lncRNAs, patients with STAD were clustered into two clusters (clusters I and II), which had significant differences in the OS rate and tumor grade. Then, 14 of 19 m^6^A-related prognostic lncRNAs were used to establish a prognostic signature with the LASSO method in the training group. Kaplan-Meier analysis showed that the OS of patients with low-risk scores was longer than those of patients with high-risk scores. Additionally, the result of ROC curve analysis indicated that the 14-lncRNAs signature could serve as a highly specific and sensitive prognostic survival model in STAD. Moreover, the results were further validated in the test groups and in the combined group. This signature can be used as an independent prognostic factor for STAD, suggesting that these 14 lncRNAs may be vital m^6^A-related lncRNAs and significant prognostic factors for patients with STAD. Furthermore, this m^6^A-related lncRNA prognostic model could serve as a prognostic indicator for OS in subgroups of patients with different clinical characteristics, especially age, sex, tumor grade, clinical stage, stages T3–T4, stage M0, and stage N.

Moreover, different clusters or subgroups were correlated with diverse TME scores, immune infiltration cells, and ICGs expression, which suggested that m^6^A-related lncRNAs may play a critical regulatory role in the TME and immune exhaustion of STAD. The tumor purity of cluster II that was associated with poorer OS and the high-risk group decreased, which indicated the promotion role of stromal cells and the dysfunction of immune infiltration cells in STAD. Fibroblasts, the most abundant stromal cell, could promote tumor proliferation and metastasis by secreting cytokines, growth factors, and chemokines and the remodeling of extracellular matrix (ECM) (48). Concerning immune infiltration cells, there is a significant positive correlation between M2 macrophages and cluster II and the high-risk group, which implicated the potential regulatory role of m^6^A-related lncRNAs in the polarization of tumor-associated macrophages. M2 macrophages could facilitate tumor occurrence and metastasis through the inhibition of T-cell-mediated antitumor immune response and the promotion of tumor angiogenesis (49). Although abundant immune cells infiltrated in TME, they could not lead to the antitumor immune response because of immune checkpoints blockade and the effects of hypoxia and metabolic alternation. To be more specific, hypoxia is strongly linked to immunosuppression *via* immune trafficking, angiogenesis, and alteration of molecular markers, which has hugely detrimental effects on T-cell function (50). Similarly, metabolic alternation also can lead to T-cell suppression, which was related to a host of metabolic byproducts (51). Moreover, as our results showed, some prominent ICGs, such as PDCD1LG2, HAVCR2, and ICOS, were significantly overexpressed in cluster II and the high-risk group, which resulted in immune exhaustion in TME. In addition, we discovered the difference in the response to ICIs between subgroups. Together, these findings suggest that the m^6^A-related lncRNAs may play a potential influence on the dysfunction of immune infiltration cells and the response to immunotherapy in STAD.

To provide a comprehensive analysis of m^6^A-related lncRNAs, a ceRNA network consisting of two lncRNAs, 24 miRNAs, and 70 mRNAs was constructed, and differentially expressed genes between different clusters or subgroups were identified for viewing the latent functions of m^6^A-related lncRNAs. With the two key m^6^A-related lncRNAs found in STAD, lncRNA MAGI2-AS3 has been reported to be associated with patient clinical characteristics in different tumors and can regulate a variety of biological processes (52). MAGI2-AS3 significantly promoted GC progression and migration *via* maintaining ZEB1 overexpression by sponging miR-141/200a (53). KEGG pathway and GO analysis shows that 70 target mRNAs and differentially expressed genes between different clusters or subgroups were enriched in several biological processes and pathways associated with the occurrence and progression of STAD (54–56), including “MAPK signaling pathway,” “PI3K-Akt signaling pathway,” “Wnt signaling pathway,” “focal adhesion,” and so on. Genes in different clusters or subgroups were functionally annotated using GSEA. Genes in cluster II and the high-risk group were also enriched in the cancer-related pathways. In addition, it should be noted that several biological processes and pathways associated with immune response were identified, such as “antigen binding”, “immunoglobulin receptor binding”, and “complement and coagulation cascades”. Previous studies have demonstrated that the activation of the complement system and coagulation cascades plays an important role in cancer malignant biological behavior (57–59).

## Conclusion

In this study, we identified two clusters based on 19 m^6^A-related lncRNAs and constructed a prognostic signature comprising 14 m^6^A-related lncRNAs in STAD, which had significant value in predicting the OS of patients with STAD, clinicopathological characteristics, TME, ICGs expression, and the response to ICIs. Additionally, biological processes and pathways associated with m^6^A-related lncRNAs were identified, which improved our understanding of the role of m^6^A-related lncRNAs in the occurrence and progression of STAD. This work also provides important evidence for the development of predictive biomarkers and immunotherapy for STAD.

## Data Availability Statement

Publicly available datasets were analyzed in this study, which can be found in the Cancer Genome Atlas (TCGA) database.

## Ethics Statement

This study met the publication guidelines stated by TCGA (https://cancergenome.nih.Gov/publications/publicationguidelines). All data used in the study were obtained from TCGA. Ethics approval and informed consent were not required.

## Author Contributions

YH, PL, and FL conceived and designed the study. YH, ZY, CH, XJ, and YY organized the database and performed statistical analyses. YH and YW wrote the first draft of the manuscript. ZY, CH, and XJ prepared the figures and tables and were involved in manuscript writing. KZ, YW, PL, and FL revised and proofread the manuscript. All authors contributed to the article and approved the submitted version.

## Funding

This study was supported by The National Natural Science Foundation (No. 81904139, No. 81973819, and No. 81904145), Collaborative Innovation Team Project of First-Rate Universities and Disciplines and High-level University Discipline of Guangzhou University of Chinese Medicine (No. 2021xk47), The Natural Science Foundation of Guangdong Province (No. 2019A1515011145), Guangdong Medical Science and Technology Research Fund (No. B2021089, No. A2020186), Clinical Research Project of Innovation Hospital in the First Affiliated Hospital of Guangzhou University of Chinese Medicine (No. 2019IIT19), and High-level Hospital Construction project of the First Affiliated Hospital of Guangzhou University of Chinese Medicine (No. 2019QN01).

## Conflict of Interest

The authors declare that the research was conducted in the absence of any commercial or financial relationships that could be construed as a potential conflict of interest.

## Publisher’s Note

All claims expressed in this article are solely those of the authors and do not necessarily represent those of their affiliated organizations, or those of the publisher, the editors and the reviewers. Any product that may be evaluated in this article, or claim that may be made by its manufacturer, is not guaranteed or endorsed by the publisher.
